# Chronic nonscarring scalp folliculitis responding to topical phosphodiesterase-4 inhibitor roflumilast

**DOI:** 10.1016/j.jdcr.2024.11.009

**Published:** 2024-11-26

**Authors:** Ekaterina Korytnikova, Charles L. Halasz

**Affiliations:** a Department of Medicine, Norwalk Hospital, Norwalk, Connecticut; b Department of Dermatology, Columbia University Irving Medical Center, New York, New York

**Keywords:** chronic nonscarring scalp folliculitis, phosphodiesterase-4 inhibitor, roflumilast

## Introduction

Chronic nonscarring scalp folliculitis (CNSSF) is an underreported but relatively common variant of scalp folliculitis that was first described in 1979 by Hersle et al.^1^ It typically affects the occipital area of the scalp and manifests as follicular acneiform pustules or papulopustules that clear up without leaving scars.^1^^,^^2^ This condition predominantly affects men and involves neutrophilic inflammation of the pilosebaceous unit.^1^, ^2^, ^3^, ^4^ Microbiological cultures typically reveal resident flora including Staphylococcus species, Propionibacteriae, occasionally Malassezia or Demodex, and uncommonly Staphylococcus aureus.^1^, ^2^, ^3^, ^4^, ^5^

The differential diagnosis of scalp folliculitis includes infectious and noninfectious folliculitis, as well as perifolliculitis. These conditions can result in either cicatricial, or noncicatricial alopecia as observed in CNSSF.^6^ In the management of CNSSF, topical treatments are generally ineffective.^1^ Systemic antibiotics often achieve lesion clearance, but relapses are frequent upon discontinuation of treatment.^1^^,^^2^

We present a case of a middle-aged male with occipital scalp nonscarring folliculitis that responded to oral antibiotics but recurred upon tapering. Near-complete resolution was achieved with the topical phosphodiesterase-4 (PDE-4) inhibitor, roflumilast. This case highlights a promising topical treatment option for refractory CNSSF, particularly significant for patients who cannot tolerate or prefer to avoid systemic medications.

## Case presentation

A 50 year old Hispanic male with native South American Indian and European ancestry and with a history of prediabetes managed through dietary and lifestyle modifications, presented with a complaint of mildly pruritic scalp pustules that had persisted for 1 year despite initial treatment with amoxicillin-clavulanate prescribed by his primary care physician. A skin examination revealed folliculitis on the nuchal-occipital and also vertex scalp without scarring.

**Fig 1:**
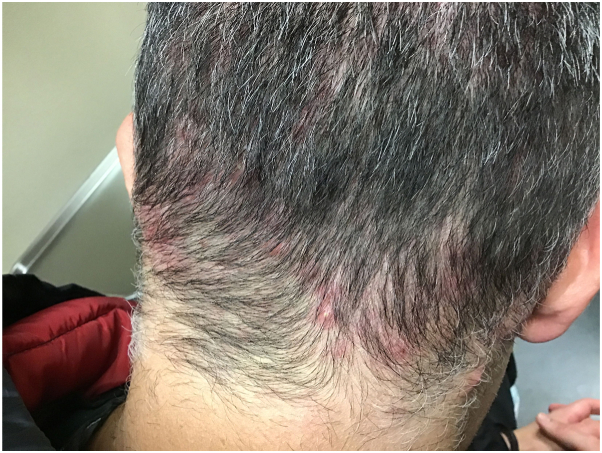
Chronic nonscarring scalp folliculitis affecting the occipital area.

Patient was diagnosed with nonscarring scalp folliculitis clinically (Fig 1). Incidental seborrheic dermatitis was noted behind the ears. Swabs of pustules submitted for aerobic culture were negative. A smear was also negative for Demodex or Malassezia.

The patient was initially treated with various topical agents, including selenium sulfide suspension, mometasone solution, mupirocin ointment, clindamycin solution, and minocycline foam, but experienced no meaningful improvement. For the treatment of recalcitrant folliculitis, the patient was switched to oral antibiotics and responded well to oral doxycycline and better to oral minocycline. However, multiple attempts to taper off antibiotics failed, and elevated liver function tests while on minocycline necessitated the discontinuation of systemic antibiotics, leading to a recurrence of the eruption.

**Fig 2:**
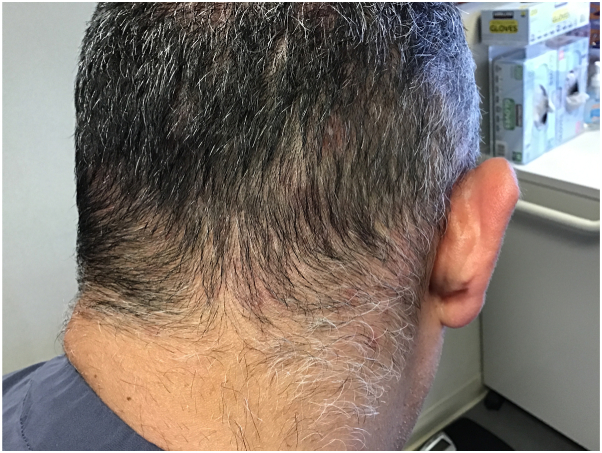
Near-complete resolution of CNSSF at 12 weeks follow-up with once daily application. *CNSSF*, Chronic nonscarring scalp folliculitis.

**Fig 3:**
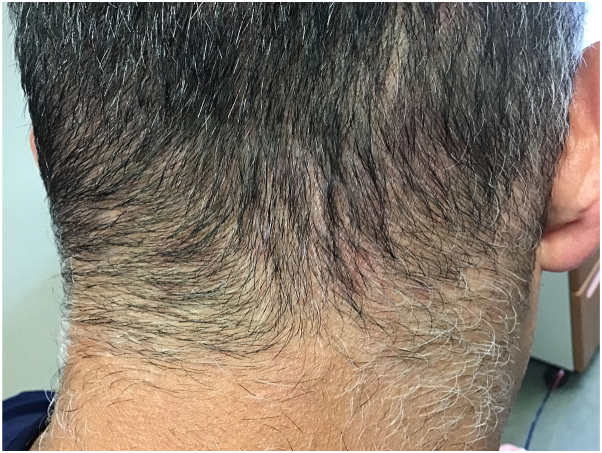
Sustained improvement of CNSSF observed at 24 weeks after tapering to application every 2-3 days. *CNSSF*, Chronic nonscarring scalp folliculitis.

Subsequently, in view of having mild seborrheic dermatitis, patient was started on roflumilast 0.3% topical foam once daily, which resulted in significant improvement of not only his seborrheic dermatitis, but also of his scalp folliculitis within 10 days. At that time, the patient had been off minocycline for 2 months. At 12-week follow-up, only 1-2 active folliculitis lesions were present on the occipital scalp, and the remainder of the scalp was clear (Fig 2). The patient remained clear at the 24-week follow-up after reducing the application frequency to every 2-3 days. (Fig 3).

## Discussion

We present a case of a middle-aged male with CNSSF responding to, but recurring after discontinuation of, oral antibiotics, and achieving fast, essentially complete resolution after treatment with the topical PDE-4 inhibitor roflumilast.

CNSSF is a subtype of scalp folliculitis characterized by recurrent follicular pustules, primarily affecting the occipital region of the scalp in 80% of cases, which heal without residual fibrosis.^2^ It is a frequently encountered condition in clinical practice, accounting in one study for 15.4% of annual hair and scalp-related visits.^7^ The most common age of onset is between 20 and 40 years, with a male predisposition.^1^, ^2^, ^3^, ^4^ The condition has been associated with an oily scalp.^1^^,^^3^

Histopathologic findings reveal neutrophilic folliculitis in the superficial aspect of the hair follicle with a perivascular lymphocytic infiltrate with eosinophils, and foreign body giant cell lymphocytes and macrophages in some case.^3^ Immunohistochemistry demonstrates strong interleukin 1 beta activity in skin-infiltrating immune cells, particularly macrophages.^4^

Clinical response to antistaphylococcal antibiotics has been effective even in the absence of Staphylococcus aureus in cultures.^2^^,^^8^ The therapeutic effect is likely attributed to the anti-inflammatory properties of the antibiotics.^2^^,^^8^ Other reported treatment modalities with anti-inflammatory effects include isotretinoin, tumor necrosis factor alpha inhibitors, and antifungal medications.^1^^,^^2^^,^^4^^,^^5^ However, relapse is frequently observed upon discontinuation of treatment, necessitating ongoing maintenance therapy.^1^^,^^2^^,^^5^

Due to the recalcitrant nature of the disease, safe and effective therapy is essential. Systemic treatments face challenges such as rising antibiotic resistance, multiple drug interactions that restrict their use in patients with multiple comorbidities, and intolerance to systemic medication side effects. However, previously explored topical treatment options for chronic nonscarring scalp folliculitis, such as alcohol-containing scalp lotions, topical corticosteroids, and topical clindamycin gel, generally do not achieve the desired therapeutic outcomes.^1^ As such, among the 3 largest case series reported, findings regarding topical treatment were either unsatisfactory,^1^ or topical therapy was not considered at all.^2^ A single study suggested topical antifungals reduced symptoms.^5^

In our case, the patient was treated with roflumilast 0.3% topical foam once daily and achieved nearly complete clearance of scalp folliculitis within 10 days. Roflumilast is a once-daily topical medication available in various formulations, including a 0.3% concentration approved for treating plaque psoriasis and seborrheic dermatitis, and a 0.15% concentration recently approved for atopic dermatitis.^9^ It inhibits PDE-4, leading to the downregulation of multiple inflammatory mediators including several key cytokines involved in neutrophil activation and chemotaxis such as tumor necrosis factor alpha, interleukin 6, and interleukin 8.^4^^,^^10^ The effect of topical roflumilast on CNSSF may therefore be due in part to its immunomodulatory impact, reducing neutrophilic inflammation.^4^

In conclusion, the topical PDE-4 inhibitor roflumilast offers a promising treatment option for patients who have exhausted other topical therapies and prefer to avoid or cannot tolerate systemic treatments. Further comprehensive studies are needed to confirm the overall efficacy of roflumilast in treating CNSSF and other scalp folliculitis conditions.

## Conflicts of interest

None disclosed.
